# Integratedly analyzed quantitative proteomics with transcriptomics to discover key genes via *fg-1* non-heading mutant in the early heading stage of Chinese cabbage

**DOI:** 10.3389/fpls.2024.1467006

**Published:** 2024-10-17

**Authors:** Jingrui Li, Mi Fan, Xiaomeng Zhang, Liling Yang, Guangguang Hou, Lei Yang, Na Li, Shuxin Xuan, Jianjun Zhao

**Affiliations:** Collaborative Innovation Center of Vegetable Industry in Hebei, Hebei Key Laboratory of Vegetable Germplasm Innovation and Utilization, College of Horticulture, Hebei Agricultural University, Baoding, Hebei, China

**Keywords:** *Brassica rapa*, leaf heading, *fg-1* EMS mutant, quantitative proteomics techniques, integration analysis

## Abstract

Leaf heading is an important agronomic trait of Chinese cabbage, which directly affects its yield. Leaf heading formation in Chinese cabbage is controlled by its internal genotype and external environmental factors, the underlying mechanism of which remains poorly understood. To discover the leaf heading formation mechanism more deeply, this study analyzed the correlation between proteomic and transcriptomic data in the leaf heading formation mutant *fg-1* generated by EMS. iTRAQ-based quantitative proteomics techniques were performed to identify the protein expression profiles during the key periods of the early heading stage in the section of the soft leaf apical region (section a) and the whole leaf basal region (section d). We first identified 1,246 differentially expressed proteins (DEPs) in section a and 1,055 DEPs in section d. Notably, transcriptome–proteome integrated analysis revealed that 207 and 278 genes showed consistent trends at the genes’ and proteins’ expression levels in section a and section d, respectively. KEGG analyses showed that the phenylpropanoid biosynthesis pathway was enriched in both sections a and d. Furthermore, 86 TFs exhibited co-upregulation or co-downregulation, and seven out of 86 were involved in plant hormone synthesis and signal transduction pathways. This indicates that they are potentially related to the leaf heading formation in Chinese cabbage. Taken together, we have identified several key early-heading-formation-related factors via integration analysis of the transcriptomics and proteomics data. This provides sufficient gene resources to discover the molecular mechanism of leaf heading formation.

## 1 Introduction

Chinese cabbage (*Brassica rapa* ssp. *pekinensis*) is an important leafy vegetable crop with a leafy head that provides abundant mineral nutrients, crude fiber, and vitamins for human diet, is a popular vegetable in East Asia, particularly in China, Korea, and Japan, and has become a worldwide vegetable crop (Ramchiary et al., 2011; Park et al., 2020). The leafy head is an essential agronomic trait to evaluate the yield and quality of Chinese cabbage. The life cycle of Chinese cabbage undergoes nine stages: germination, seedling, rosette, folding, heading, post-heading, bolting, flowering, and podding (Yu et al., 2000; Wang et al., 2011). After the rosette stage, the edges of new leaves curl inward, the leaf angle decreases, and the leafy head forms (Li et al., 2019b). Heading formation is an extremely complex biological process in many leafy vegetables such as lettuce and cabbage. Numerous researchers have attempted to elucidate the molecular mechanisms of leaf heading formation related to morphology and genetics (Tabusam et al., 2022; Cheng et al., 2016; Yu et al., 2020; Alemán-Báez et al., 2022).

The basis of leafy head formation is leaf development. Currently, research on the leaf development mechanism of the model plant *Arabidopsis* is the most in-depth. Leaf-polarity-related genes have been identified, and the regulatory network is relatively clear (Ali et al., 2020). The related research results have laid the foundation for the study of leafy head development in Chinese cabbage. Chinese cabbage, also a member of the Brassicaceae family, has curled leaves and forms leafy heads with different embracing models, which is a specific trait for some *Brassica* species but not for *Arabidopsis* species. Moreover, the Chinese cabbage genome has undergone a triploid replication process, so the molecular regulatory mechanism of leaf development, especially leafy head development, is much more complex than that of *Arabidopsis* (Wang et al., 2011). The He Yuke Laboratory of the Chinese Academy of Sciences was the first to confirm at the molecular level that plant auxin genes are involved in regulating the formation of Chinese cabbage leaf heading (He et al., 2000; Yu et al., 2000, 2013). The whole-genome sequence analysis of Chinese cabbage chiifu401 identified the organ morphology and development-related genes (including auxin synthesis, transport, signal transduction, etc.) that may affect leaf heading development (Wang et al., 2011; Cheng et al., 2016). Using used 199 Chinese cabbage (*B. rapa*) with leaf heading and 119 cabbage (*B. oleracea*) non-heading genotypes as materials to reveal leaf heading development by genomic resequencing showed that leaf heading development is related to hormone and dorsoventrality-related genes as well as small-molecule-RNA (miRNA) gene.

It is generally believed that transcriptional profiling provides an overview of the potential functions of genes (Zhang et al., 2017). At the transcriptional level, the mechanism of leafy head formation has been preliminarily analyzed (Wang et al., 2012; Sun et al., 2019). However, most biological functions are carried out by proteins, and detecting changes in protein expression is thus more valuable than detecting changes in gene expression. Quantitative proteomics techniques, isobaric tags for relative absolute quantitation (iTRAQ)/tandem mass tag (TMT), have been used to analyze biological processes (Li et al., 2022; Liu et al., 2023) and leafy head formation (Zhang et al., 2016). Although transcriptomic and proteomic analyses are independent, a gene can control the generation of several proteins, and the synthesis of a protein may also involve the transcription of multiple genes. Therefore, gene transcription or protein translation alone cannot fully reflect the regulatory network of gene expression during plant development. In recent years, the integration of transcriptomics and proteomics has gradually become an important strategy to study the mechanisms of biological processes and the responses to various abiotic and biological stresses (Zhang et al., 2018; Ding et al., 2020). However, few studies have analyzed leafy head formation through a method integrating transcriptomics and proteomics (Zhang et al., 2016).

Leafy head formation, a quantitative trait additively controlled by low-dominance effects, is a complex biological process affected by many factors such as hormones, temperature, light, and so on (Ito and Kato, 1957; Tanaka et al., 2009). Exogenous GA_3_ treatment leads to the non-heading mutant plant phenotype restored to the heading of the wild type of Chinese cabbage (Gao et al., 2020), and low temperature induced leaf heading formation (Zhang et al., 2016). Notably, former studies verified that the leafy-head-formation-related genes normally used different heading types of natural population materials, but their genetic backgrounds are too complex to identify the key genes efficiently. Besides that, most of the studies identify the related genes at the transcriptional level, which cannot fully refer to the real regulated functions of proteins. Therefore, in the present study, we selected flat growth mutant (*fg-1*), which was obtained from the EMS mutant library constructed in our laboratory (Lu et al., 2016b). Integratedly analyzed quantitative proteomic data obtained using the iTRAQ-based quantitative proteomics with transcriptome data previously obtained by RNA-Seq (Li et al., 2019a) was used to identify the candidate pathways and genes related to leaf heading formation in *fg-1*-mutant Chinese cabbage at the early leaf heading stage.

Leafy head formation is a complex biological process affected by many factors. Research showed that leaf heading formation in Chinese cabbage is a quantitative trait controlled additively by low-dominance effects that can be affected by auxin concentration, temperature, light intensity, and the ratio of carbohydrate to nitrogen among other factors (Ito and Kato, 1957; Tanaka et al., 2009). However, other research showed that the leaf heading trait is controlled by a pair of recessive alleles (Li et al., 2019a; Gao et al., 2020). Nowadays, an increasing number of studies have shown that plant hormones play important roles in leafy head formation (Gao et al., 2017; Gu et al., 2017; Yue et al., 2022). To identify the genes related to leafy head formation, former studies normally used Chinese cabbage of different heading types as materials, but the genetic backgrounds of the materials were not consistent. Besides that, most studies mainly focused on transcriptomic analysis but not proteomic analysis, in which genes-to-transcripts cannot fully explain the systemical function of proteins *in vivo*. Using transcriptomics and proteomics integrating analysis would be a more efficient assay to study leaf heading formation. Therefore, in the present study, we integratedly analyzed quantitative proteomic data obtained using the iTRAQ-based quantitative proteomics with transcriptome data previously obtained by RNA-Seq (Li et al., 2019a) to identify the candidate pathways and genes related to leaf heading formation in *fg-1*-mutant Chinese cabbage at the early leaf heading stage, which was obtained from the EMS mutant library constructed in our laboratory and exhibited flat growth (Lu et al., 2016b). These results provided abundant candidate genes and key pathways and contributed deeper insights into the underlying molecular mechanisms of leaf heading formation.

## 2 Materials and methods

### 2.1 Plant materials

*fg-1*-mutant and WT lines were used as the experimental materials for the proteomic analysis of the mutant trait. The WT and *fg-1*-mutant seeds were sown on 50-hole trays in the plastic tunnel on the experimental farm at Hebei Agricultural University in Baoding (115.47 E, 38.87 N), China. Grown for 25 days, the seedlings were planted in soil in the greenhouse. Afterward, routine management was carried out. At 55 days after planting, proteome sampling was conducted, and three biological replicates were used for analysis. All leaf samples were frozen in liquid nitrogen to extract RNA immediately or stored at -80°C.

### 2.2 Protein extraction, iTRAQ labeling, and LC−MS/MS analysis

Extraction of protein from the sample was done by grinding, cracking, precipitation, washing, and redissolving according to a previous method (Zeng et al., 2018), and the protein concentration was determined by using a BCA kit (Beyotime Biotechnology) according to the manufacturer’s instructions. The proteins were then subjected to trypsin digestion and labeled with the iTRAQ 8-plex kits (Applied Biosystems) according to the manufacturer’s instructions. The tryptic peptides were fractionated by high-pH reverse-phase HPLC using an Agilent 300Extend C18 column.

LC−MS/MS analysis was performed on an EASY-nLC 1000 UPLC system. The peptides were subjected to NSI source followed by tandem mass spectrometry (MS/MS) in a Q ExactiveTM Plus instrument (Thermo) coupled online to the UPLC system.

### 2.3 Protein identification and quantification

The resulting MS/MS data were processed using the MaxQuant search engine (v.1.5.2.8). Tandem mass spectra were searched against the http://brassicadb.org/brad/database concatenated with the reverse decoy database. The FDR for protein identification and peptide spectrum match identification is set to 1%. Proteins with fold change >1.2 and *p*-value <0.05 in the two samples were considered as significant DEPs.

### 2.4 Bioinformatics methods

The Gene Ontology (GO) annotation proteome was derived from the UniProt-GOA database (www.http://www.ebi.ac.uk/GOA/). The Kyoto Encyclopedia of Genes and Genomes (KEGG) (http://www.genome.jp/kegg/) database was used for the annotation of protein pathways. The identified protein domain functional descriptions were annotated with InterProScan (a sequence analysis application) based on the protein sequence alignment method, and the InterPro domain database (http://www.ebi.ac.uk/interpro/) was used. The following criteria were used to determine the significant enrichment of GO terms, KEGG pathways, and protein domains: 1.2-fold change and a *p*-value ≤0.05.

### 2.5 Analysis of the correlations between the proteomic and transcriptomic results

The transcriptomic data used in this study were obtained from a previous study (Li et al., 2019a). By comparing the quantitative results of transcriptome and proteome, the genes that were quantified at both transcriptome and proteome levels were found. The correlation analysis was performed with R (version 3.5.1), and scatter plots were drawn based on the changes in the transcriptomic and proteomic results. When log2 FC >0 and the verified *p*-value <0.05, it is a significantly differentially expressed upregulated transcript; when log2 FC <0 and the verified *p*-value <0.05, it is a significantly differentially expressed downregulated transcript. When the ratio is greater than 1.2 and the *p*-value is less than 0.05, it indicates significant differential expression of upregulated proteins. When the ratio is less than 1/1.2 and the *p*-value is less than 0.05, it indicates significant differential expression of downregulated proteins. Through the above-mentioned criteria screening, significantly differentially expressed transcripts and proteins were obtained in each comparison group. The DEGs and DEPs were separately counted, and Venn diagrams were generated according to the results. The significant enrichment of GO terms and KEGG pathways was performed on the co-upregulated and co-downregulated transcripts separately.

## 3 Results

### 3.1 DEPs’ identification of *fg-1* mutant and WT in the early heading stage

**Figure 1:**
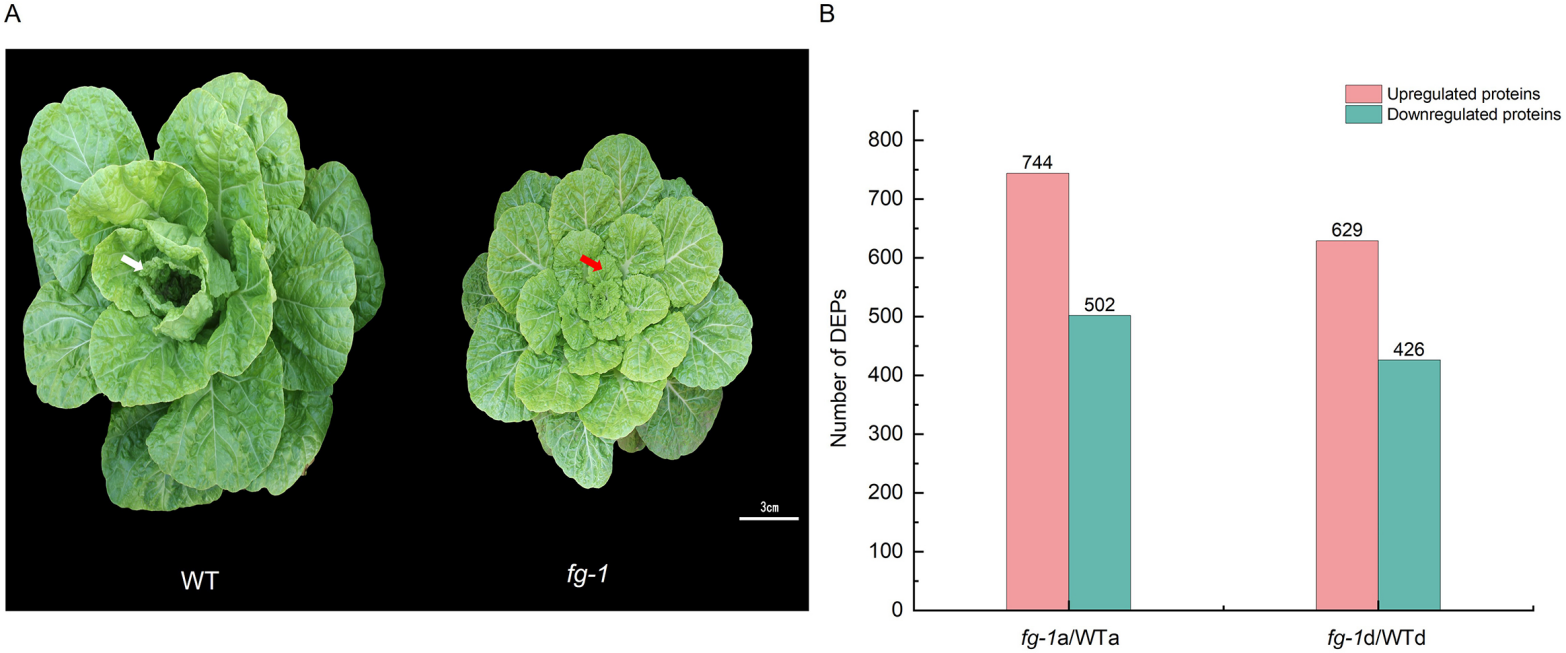
DEPs’ identification of *fg-1* mutant and WT in the early heading stage of Chinese cabbage. Wild type and mutant of Chinese cabbage growing for 55 days in the early heading stage **(A)** and differentially expressed proteins (DEPs) **(B)**. The white arrow indicates the curling leaf in WT, and the red arrow indicates the flat leaf in *fg-1* in the early heading stage. Scale bar = 3 cm.

The flat growth mutant (*fg-1*) of the M6 generation was obtained from the mutant library of Chinese cabbage, which was previously developed by EMS treatment of seeds from the inbred wild-type (WT) line (Lu et al., 2016b). The *fg-1* mutant has flat leaves during growth before the heading stage and trends to exhibit heading at the heading stage, and WT exhibits an outward-curling heading pattern. Throughout the entire growth period of Chinese cabbage, the mutant and wild type are significantly different, especially in the early stage of heading. The leaf apical region of wild-type Chinese cabbage begins to embrace inward, and the leaf angle (the angle between the blade and the center) becomes smaller to form the leaf heading, while the mutant leaves continued with their flat growth until the harvest period (**Figure 1A**). Our previous study showed that the phenotypic traits of F1 plants were similar to the wild type, and the segregation ratio of the F2 plants’ traits conformed to the 3:1 ratio. The genetic analysis shows that the mutant trait is controlled by a pair of recessive alleles, while it is unknown which gene regulates leafy head (Li et al., 2019a).

To verify the key regulatory factors of leafy head formation, the proteome assays were first performed in the *fg-1* mutant and WT. In the early heading stage of *fg-1* mutant and WT (55 days after planting), the 16th leaf from the exterior of the leafy head was sampled at two positions—apical to the leaf (a) and basal to the leaf (d)—for proteome analysis (**Supplementary Figure S1**). After merging the data from three biological replicates, a total of 266,694 spectra were generated by iTRAQ of WT and *fg-1* Chinese cabbage leaves. Furthermore, 70,739 out of 266,694 spectra matched to known spectra, which identified 28,081 unique peptides and 7,287 proteins (**Supplementary Table S1**). Further analysis showed that the lengths of the peptides were mainly distributed in seven to 20 amino acids (**Supplementary Figure S2A**), and the primary mass error of the peptides was within 10 ppm (**Supplementary Figure S2B**). Besides that, the protein mass showed that the molecular weight of the protein was negatively correlated with the coverage (**Supplementary Figure S2C**). Next, we identified differentially expressed protein (DEP) species by exhibiting a fold change >1.2 and *p*-value <0.05. As the results have shown, a total of 1,246 DEPs (744 with increased levels and 502 with decreased levels) and 1,055 DEPs (629 with increased levels and 426 with decreased levels) were identified in section a and section d, respectively (**Figure 1B**). These indicate that the growth defects of the *fg-1* mutant result in numerous DEPs, which might be involved in leafy head formation in the early stage.

### 3.2 DEPs’ functional characterization of *fg-1* mutant and WT in the early heading stage

**Figure 2:**
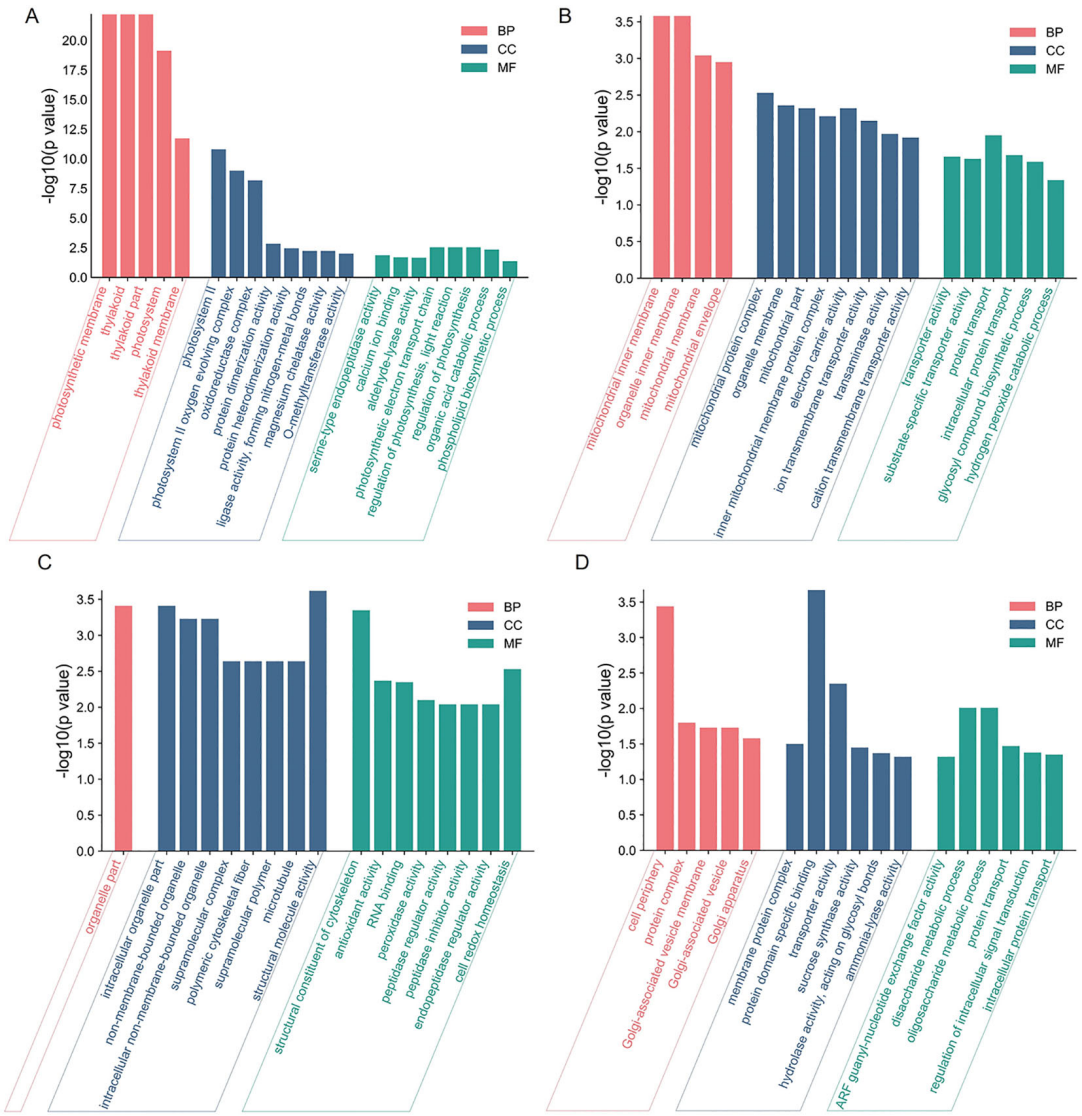
GO enrichment of DEPs in *fg-1* and WT. GO enrichment analysis of upregulated DEPs **(A)** and downregulated DEPs **(B)** in section a and upregulated DEPs **(C)** and downregulated DEPs **(D)** in section d. The DEPs were assigned to the three GO categories: biological processes (BP), cell components (CC), and molecular functions (MF). The X-axis represents the GO functional classification, and the Y-axis shows -log10 (*p*-value) enriched for each term.

To further characterize the features of DEPs on biological processes (BP), molecular functions (CC), and cellular composition (MF), GO enrichment analyses were conducted. Most of the upregulated DEPs were involved in photosynthetic-related BP and CC GO terms, such as “photosynthetic electron transport chain” and “photosynthetic membrane” in section a (**Figure 2A**); downregulated DEPs were involved in “protein transport”, “mitochondrial inner membrane”, and “electron carrier activity” in section d (**Figure 2B**). Upregulated DEPs were involved in “cell redox homeostasis”, “organelle part”, and “structural molecule activity” in section d (**Figure 2C**), and downregulated DEPs were involved in “disaccharide metabolic process”, “cell periphery”, and “protein domain specific binding” in section d (**Figure 2D**). These results suggested that “regulation of photosynthesis”, “organic acid catabolic process”, “protein transport”, and “cell redox homeostasis” may play important roles in leaf heading formation.

**Figure 3:**
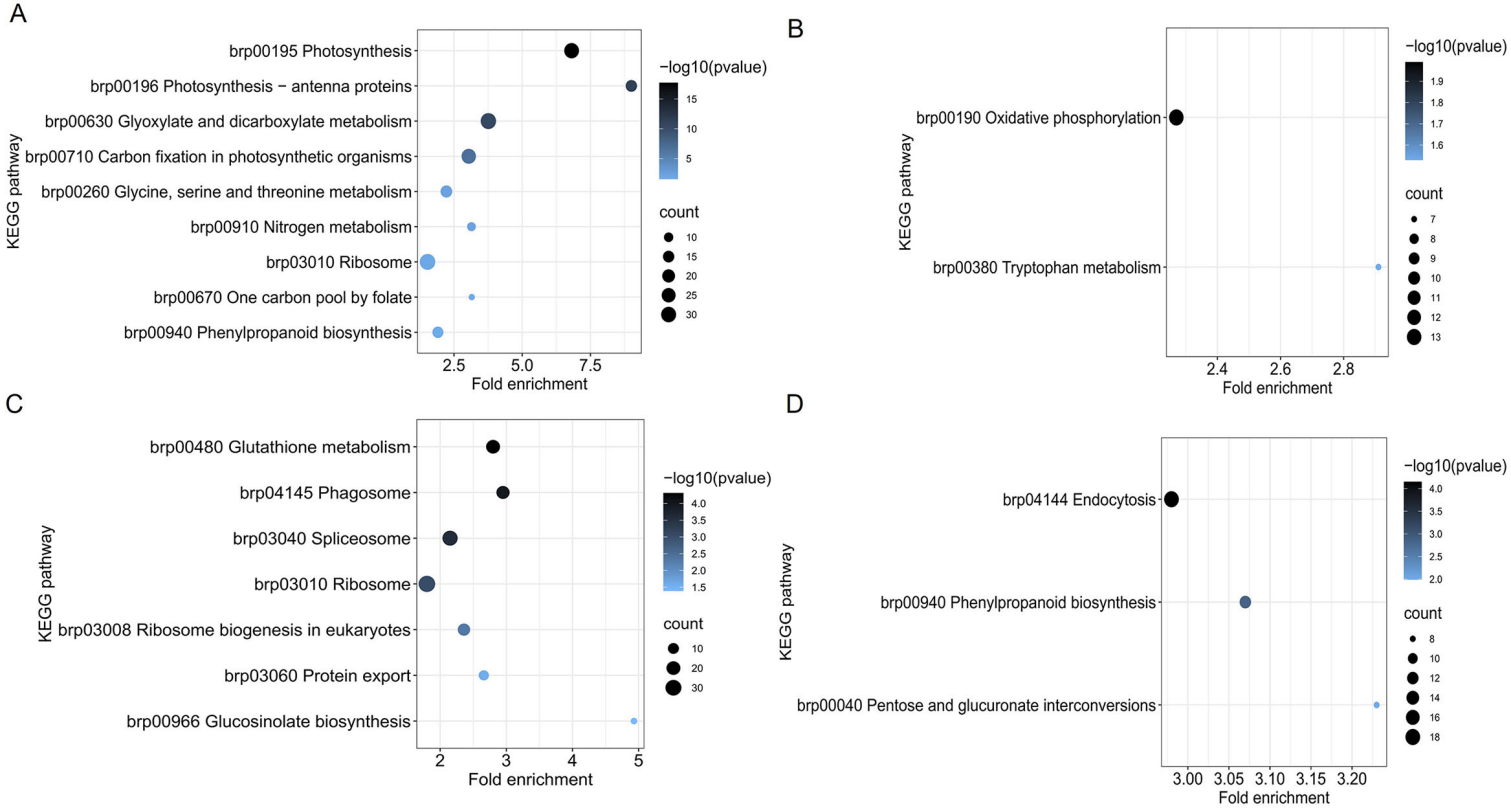
KEGG pathway enrichment of DEPs in *fg-1* and WT. KEGG pathway analysis of upregulated expressed proteins **(A)** and downregulated proteins **(B)** in section a and upregulated expressed proteins **(C)** and downregulated proteins **(D)** in section d. The vertical axis represents the pathway, and the horizontal axis represents the fold enrichment. The circle color indicates the significance of the enrichment with a -log10 (*p*-value), and the circle size represents the number of differential proteins in the pathway.

To further investigate the function of DEPs, we analyzed KEGG pathway enrichment in some specific biological processes. There were 860 DEPs in section a of the mutant and WT, the upregulated DEPs were enriched with nine KEGG pathways, and downregulated DEPs were enriched with two KEGG pathways (**Figures 3A, B**). There were 650 DEPs in section d of the mutant and WT, the upregulated DEPs were enriched with seven KEGG pathways, and downregulated DEPs were enriched with three KEGG pathways (**Figures 3C, D**). Among the enrichment KEGG, phenylpropanoid biosynthesis (brp00940) was not only enriched in one comparison, which may play a key role in leaf heading formation.

### 3.3 Verified DEPs’ and DEGs’ association of *fg-1* mutant and WT in the early heading stage

**Figure 4:**
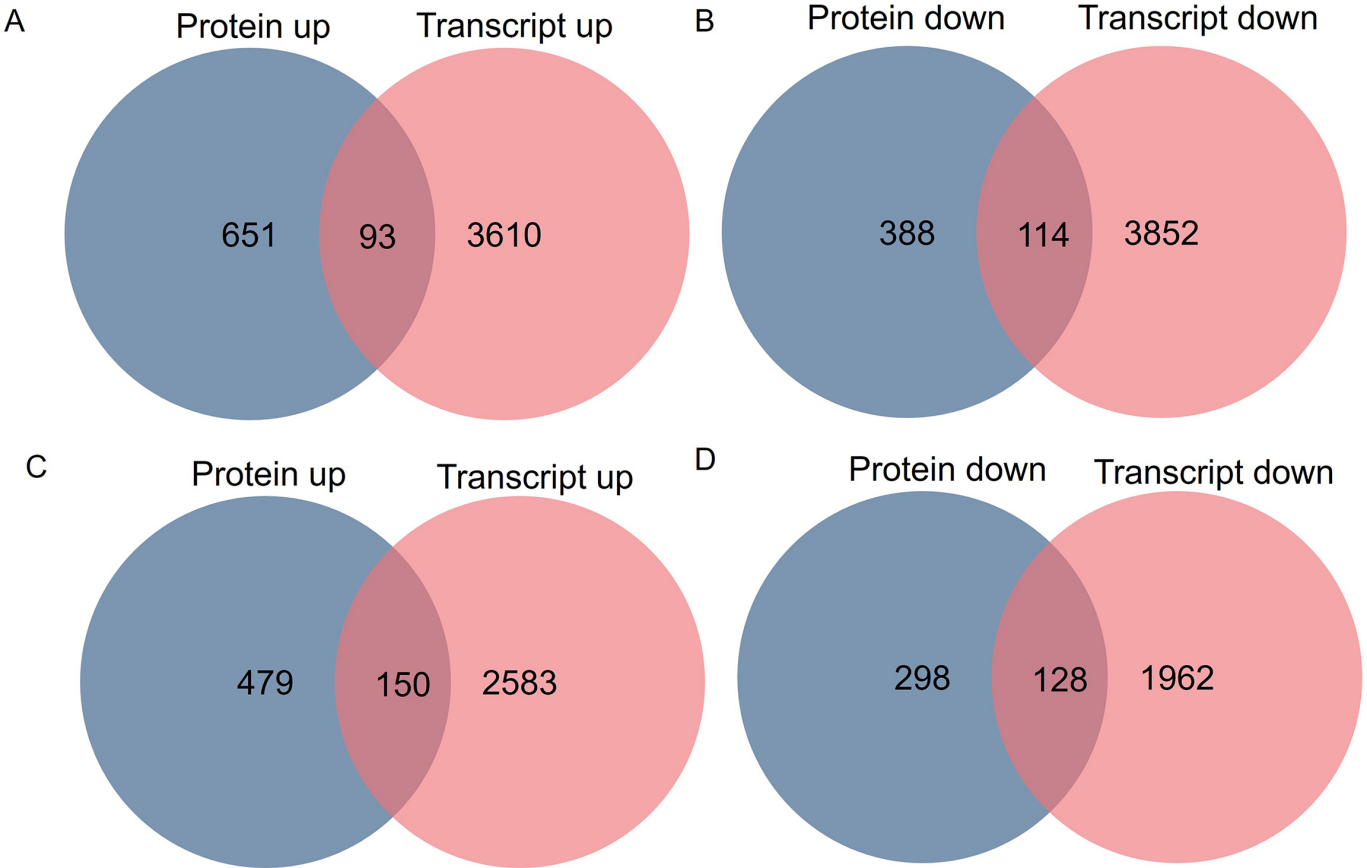
Comparative analysis of differentially expressed proteins and transcripts in *fg-1* mutant and WT. Venn diagram of upregulated genes and proteins **(A)** and downregulated genes and proteins **(B)** in section a and upregulated genes and proteins **(C)** and downregulated genes and proteins **(D)** in section d.

To study the association of proteomic and previous transcriptomic data (Li et al., 2019a), integrated analysis was performed. In section a, 7,669 mRNAs and 1,246 proteins were subjected to integrative analysis, and 272 of these were identified in both transcriptomic and proteomic analyses (**Figure 4A**). In section d, 4,823 mRNAs and 1,055 proteins were subjected to integrative analysis, and 304 of these were included in both the transcriptomic and proteomic results (**Figure 4B**). Additionally, the changes in expression at the transcript (log2 ratio of transcript) and protein (log2 ratio of protein) levels were analyzed via scatter plots. The data revealed correlations in section a (Pearson correlation coefficient, *r* = 0.40) (**Supplementary Figure S3A**) and in section d (Pearson correlation coefficient, r = 0.37) (**Supplementary Figure S3B**). Moreover, in section a, a total of 207 candidates’ protein and transcript expression levels exhibited the same up- and downregulated trends (93 upregulated and 114 downregulated) (**Figures 4A, B**). In section d, a total of 278 candidates’ protein and transcript expression levels exhibited the same up- and downregulated trends (150 upregulated and 128 downregulated) (**Figures 4C, D**).

**Figure 5:**
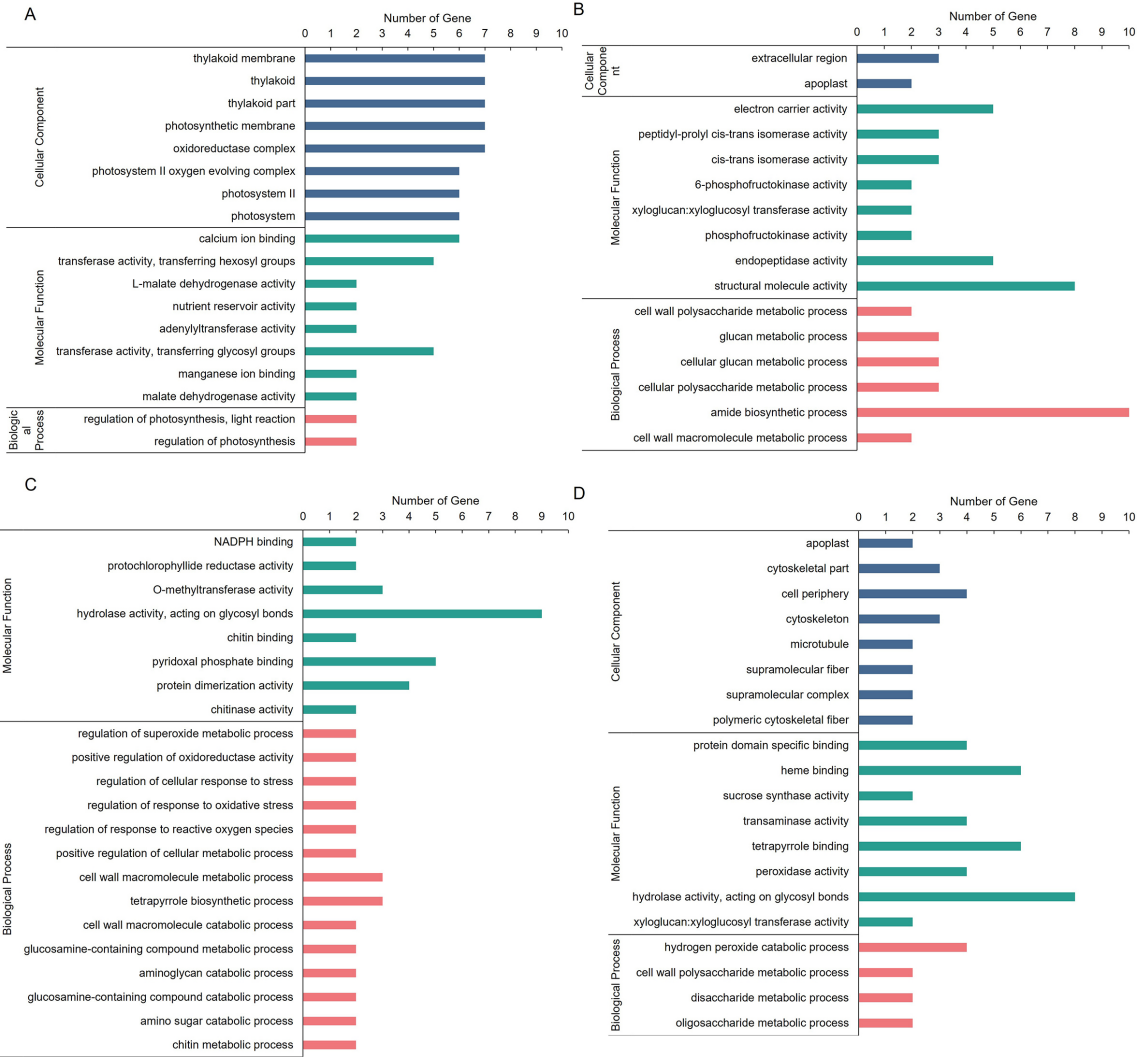
GO enrichment of DEPs in *fg-1* and WT. GO enrichment analysis of co-upregulated genes **(A)** and co-downregulated genes **(B)** in section a and co-upregulated genes **(C)** and co-downregulated genes **(D)** in section d. The DEPs were assigned to the three GO categories: biological processes (BP), cell components (CC), and molecular functions (MF). The X-axis represents the GO functional classification, and the Y-axis shows the gene numbers enriched for each term.

Next, we integratedly analyzed GO and KEGG enrichment to explore the specific biological functions of these differentially expressed proteins and transcripts. In the GO enrichment analysis, upregulated candidates in section a were annotated to 18 GO terms (**Figure 5A**), including regulation of photosynthesis, light reaction, thylakoid membrane, calcium ion binding, and so on. The downregulated candidates in section a were annotated to 22 GO terms; among them, cell wall polysaccharide metabolic process, electron carrier activity, and extracellular region were the most enrichment GO terms in BP, CC, and MF, respectively (**Figure 5B**). In addition, the upregulated candidates in section d were annotated to 16 GO terms, including cell redox homeostasis, organelle part, structural molecule activity, and so on (**Figure 5C**), and the downregulated candidates in section d were annotated to 20 GO terms, including disaccharide metabolic process, cell periphery, protein domain specific binding, and so on (**Figure 5D**). Some of the GO terms were related to photosynthesis.

**Figure 6:**
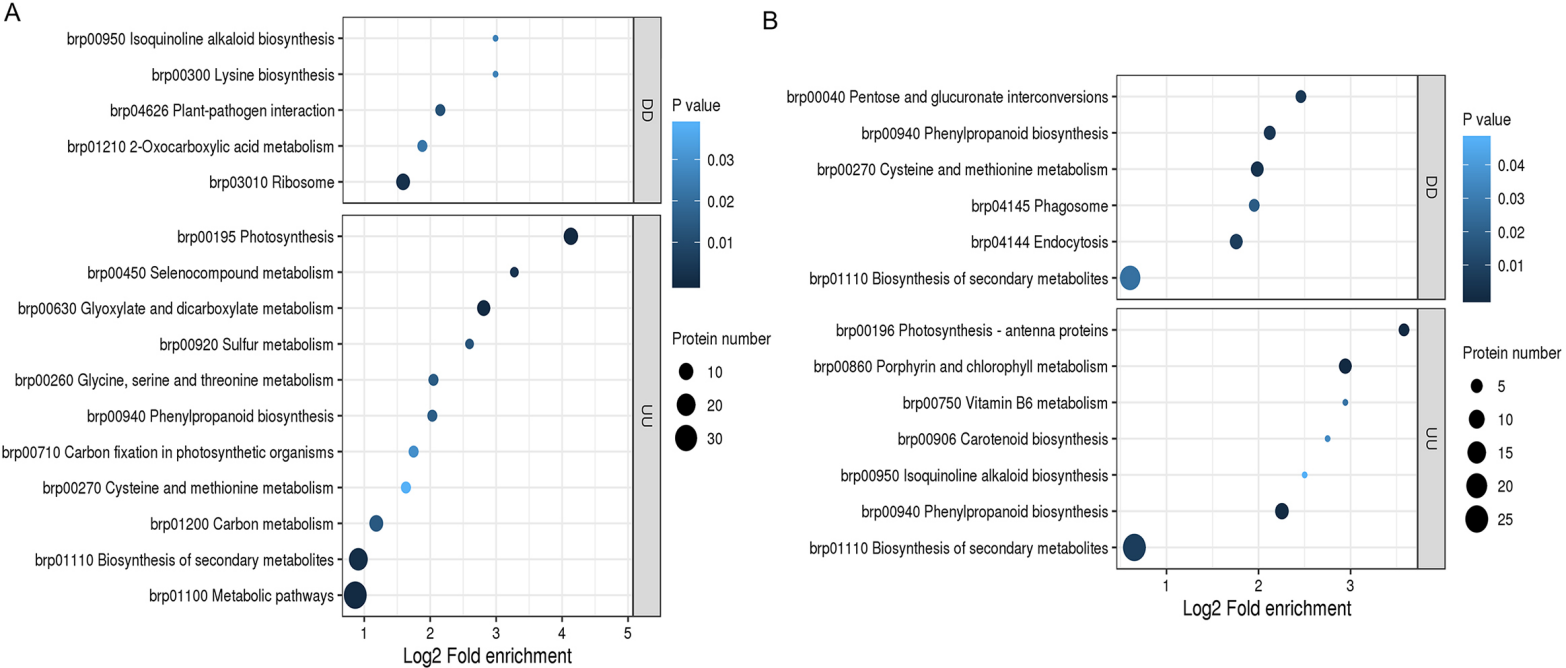
KEGG pathway enrichment of co-regulated genes in *fg-1* and WT. KEGG pathway enrichment analysis of co-regulation in section a **(A)** and section d **(B)**. DD indicates co-downregulation of genes, and UU indicates co-upregulation of genes. The horizontal axis represents the log2-transformed fold enrichment, the vertical axis represents the functional classification, the bubble size indicates the number of proteins, and the bubble color represents the enrichment significance (*p*-value).

**Figure 7:**
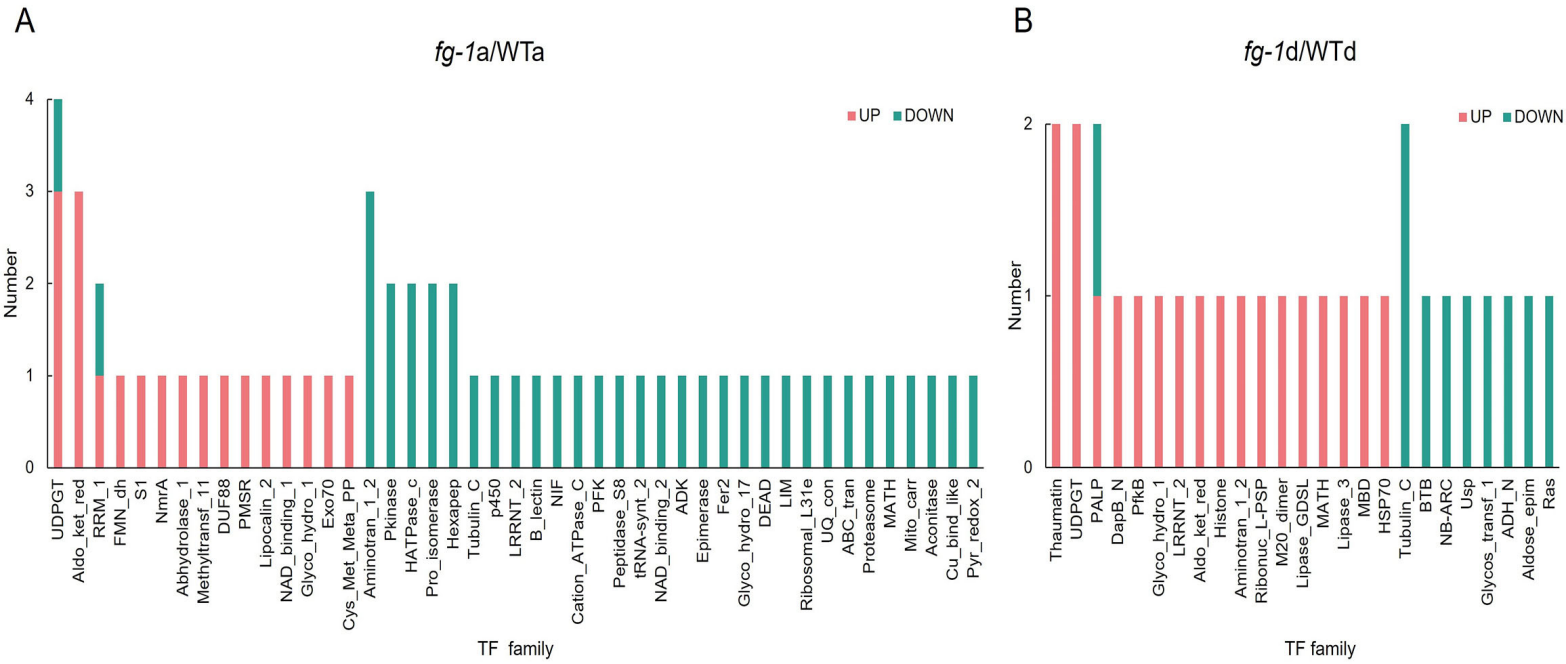
TF enrichment of *fg-1* mutant and WT. TF analysis in section a **(A)** and section d **(B)**. The x-axis represents the TF amounts, and the y-axis represents the classification. Orange represents co-upregulated factor, and blue represents co-downregulated TFs.

The upregulated transcripts and proteins were annotated to 11 KEGG pathways in section a and seven KEGG pathways in section d (**Figures 6A**, **7B**). Among them, phenylpropanoid biosynthesis (brp00940) and biosynthesis of secondary metabolites (brp01110) were enrichment pathways in sections a and d. The downregulated transcripts and proteins were annotated to five KEGG pathways in section a, including isoquinoline alkaloid biosynthesis (brp00950) (**Figure 6C**), and six KEGG pathways in section d, including phenylpropanoid biosynthesis (brp00940) (**Figure 6D**). The KEGG enrichment analysis showed that phenylpropanoid biosynthesis and biosynthesis of secondary metabolite pathway were enrichment in both sections a and d of upregulated transcripts and proteins. The pathway may be important in leaf heading formation.

### 3.4 Gene of transcription factor analysis of *fg-1* mutant and WT

Numerous studies have shown that transcription factors (TFs) are involved in regulating the leaf development of Chinese cabbage. Thus, we next performed an additional transcriptional enrichment analysis to identify key TFs. As the results showed, 86 TFs were exhibited to be co-upregulated and co-downregulated, in which 19 TFs’ transcripts/proteins were co-upregulated and 38 TFs’ transcripts/proteins were co-downregulated in section a (**Figure 7A**) and 19 TFs’ transcripts/proteins were co-upregulated and 10 TFs’ transcripts/proteins were co-downregulated in section d (**Figure 7B**). Among these, *UDPGT* (*UDP-glucosyltransferase*) has the highest number with four TF candidates, *RRM-1* and *Pkinase* have three TF members, nine transcription factors have two TFs candidates, and most of the TFs have one member only. *UDPGT* was the key regulator involved in zetin biosynthesis pathway, and *Pkinase* was the key regulator involved in signal transduction pathway. These indicates that hormone synthesis and signal transduction might play an important role in leaf heading formation in Chinese cabbage.

### 3.5 Identification of key genes and validation of gene expression levels

To further explore potential factors that regulate leaf heading formation, we analyzed the genes related to hormone synthesis and signal transduction pathways in proteomic and transcriptomic data. The analysis discovered that there were 16 genes in the plant hormone synthesis and signal transduction pathways (**Table 1**). In section a, there were six genes that were co-regulated, including *UGT73C* (*UDP-glucosyltransferase 73C*), *metB* (*cystathionine gamma-synthase*), *GOT1* (*aspartate aminotransferase, cytoplasmic*), *TAT* (*tyrosine aminotransferase*), *SIMKK* (*mitogen-activated protein kinase kinase*), and *PR1* (*pathogenesis-related protein 1*). Among these genes, except *metB* that was co-upregulated, the other genes were co-downregulated. In section d, there are 10 genes that were co-regulated, including *ZEP* (*zeaxanthin epoxidase*), two *ACO* (*1-aminocyclopropane-1-carboxylic acid oxidase*), *TAT* (tyrosine aminotransferase), *LOX2S* (*lipoxygenase2S*), *PYR/PYL* (*pyrabactin resistance 1*), *GH3* (*Gretchen Hagen 3*), *NPR1* (*non-expresser of pathogenesis-related 1*), and two *TCH4* (xyloglucosyl transferase TCH4). Among these genes, *TAT*, *LOX2S*, and *GH3* were co-upregulated, and the other genes were co-downregulated.

**Table 1:** Differentially expressed genes related to hormone synthesis and signal transduction pathways in *fg-1* and WT.

Section	Gene ID	Gene name	KEGG pathway	Hormone-related	Up/Down	TF family
Section a	Bra017224	UGT73C	Zeatin biosynthesis	Cytokinine	Down	UDPGT
	Bra039144	metB	Cysteine and methionine metabolism	Ethylene	Up	Cys_Met_Meta_PP
	Bra006103	GOT1	Cysteine and methionine metabolism	Ethylene	Down	Aminotran_1_2
			Phenylalanine metabolism	Salicylic acid		
	Bra007610	TAT	Cysteine and methionine metabolism	Ethylene	Down	Aminotran_1_2
			Phenylalanine metabolism	Salicylic acid		
	Bra014295	SIMKK	Plant hormone signal transduction	Ethylene	Down	Pkinase
	Bra013123	PR1	Plant hormone signal transduction	Salicylic acid	Down	–
Section d	Bra037130	ZEP	Carotenoid biosynthesis	Abscisic acid	Down	–
	Bra003687	ACO	Cysteine and methionine metabolism	Ethylene	Down	–
	Bra031090	ACO	Cysteine and methionine metabolism	Ethylene	Down	–
	Bra024204	TAT	Cysteine and methionine metabolism	Ethylene	Up	Aminotran_1_2
			Phenylalanine metabolism	Salicylic acid		
	Bra003526	LOX2S	α-Linolenic acid metabolism	Jasmonic acid	Up	–
	Bra021032	PYR/PYL	Plant hormone signal transduction	Abscisic acid	Down	–
	Bra006196	GH3	Plant hormone signal transduction	Auxin	Up	–
	Bra016938	NPR1	Plant hormone signal transduction	Salicylic acid	Down	BTB
	Bra024089	TCH4.1	Plant hormone signal transduction	Brassinosteroid	Down	–
	Bra011179	TCH4.2	Plant hormone signal transduction	Brassinosteroid	Down	–

## 4 Discussion

### 4.1 Identifying the key factors of plant development by iTRAQ

Proteins are the final executors of most cellular functions. Therefore, proteomics may be an effective strategy to identify the proteins or pathways that are responsible for plant development (Nogueira et al., 2012; Feng et al., 2018). The quantitative proteomics techniques have been used to analyze the ethylene-induced adventitious rooting process, and this proteomic analysis identified 24 DEPs related to adventitious root development. The enzyme SAMS (S-adenosylmethionine synthase), which is upstream of ethylene synthesis, is directly involved in adventitious root development in cucumber (Lyu et al., 2021). The proteomic profile of K326 and Honghua Dajinyuan (HD) tobacco leaves has been found to vary among the four growth stages, and the galactose metabolism and glycosphingolipid biosynthesis-globo series pathways were significantly enriched in HD at the rosette growth stage, indicating that these genes may be correlated with tobacco mosaic disease (Chen et al., 2019). However, few proteomic studies of Chinese cabbage have explored the mechanism of leaf heading formation. Leaf heading is an important economic and breeding trait of Chinese cabbage. The molecular mechanisms involved in leaf heading formation remain unclear. Early leaf heading is the key stage for leaf heading formation, and *BcpLH*, one of the key genes to regulated leaf heading formation, was cloned in the early leaf heading stage (Zhang et al., 2016, 2022; Yu et al., 2000). Our study profiled the different sections and expression patterns of proteins involved in leaf heading formation in the early heading stage via the quantitative proteomics method. The analysis identified 1,246 DEPs in section a, 1,055 DEPs in section d, and 301 DEPs in both sections a and d. The upregulated DEPs were enriched with nine KEGG pathways, and the downregulated DEPs were enriched with two KEGG pathways in section a. The upregulated DEPs were enriched with seven KEGG pathways, and the downregulated DEPs were enriched with three KEGG pathways. Phenylpropanoid biosynthesis is the enrichment in section d.

### 4.2 Key pathways are correlated with leaf heading formation

Both transcriptomic and proteomic data are important to understand the molecular processes involved in plant development (Galindo González et al., 2012; Haider and Pal, 2013; Wang et al., 2016). An analysis integrating proteomic and transcriptomic data to identify proteins and genes related to pepper genetic male sterility identified 52 DEGs/DEPs at both the proteomic and transcriptomic levels, and these DEPs and DEGs are involved in pollen exine formation, pollen wall assembly, pollen development, and phenylpropanoid biosynthesis (Cheng et al., 2019). An analysis of *Arabidopsis* seed development revealed that the proportion of genes with no correlation or even negative correlation between their protein and transcription levels increased during development, and the enriched pathways at different developmental stages differed. In the early developmental stage of *Arabidopsis* seeds, RNA processing and translation were enriched, and in the later developmental stage, photosynthesis, energy production, and metabolic processes were enriched (Mergner et al., 2020). Integrated analyses of *ask1*-mutant and wild-type *Arabidopsis* floral organs at the protein and transcription levels revealed the regulation of transcription regulators, kinases, peptidases, and ribosomal proteins, with implications for possible mechanisms of ASK1-E3 functions in floral development (Lu et al., 2016a). In our study, a total of 207 proteins exhibited the same expression patterns as did their transcripts in section a, and 278 transcripts and proteins exhibited the same trends in section d. In the KEGG enrichment analysis, the upregulated transcripts and proteins in section a were annotated to 11 KEGG pathways, including photosynthesis, and the downregulated transcripts and proteins were annotated to five KEGG pathways, including isoquinoline alkaloid biosynthesis. In section d, the upregulated transcripts and proteins were annotated to seven KEGG pathways, including photosynthesis-antenna proteins and phenylpropanoid biosynthesis, and the downregulated transcripts and proteins were annotated to six KEGG pathways, including phenylpropanoid biosynthesis. Pathways of photosynthesis-antenna proteins and phenylpropanoid may be related to leaf heading. Phenylpropanoid biosynthesis is one of the most important KEGG pathways in plants, including phenylalanine metabolism, which plays an important role in plant development and may be related to leaf heading (Dong and Lin, 2021). In BrAN3-silencing plants of Chinese cabbage, which stimulated leafy head formation at the early stage, DEGs are also enriched in the biosynthesis pathway of photosynthesis-antenna proteins and phenylpropanoid (Yu et al., 2019). These pathways play an important role in plant development and responding to phytohormones signals (Han et al., 2022; Li et al., 2023); therefore, these pathways may regulate Chinese cabbage leaf heading formation through phytohormone pathway regulation.

### 4.3 Plant hormone pathways are correlated with leaf heading formation

Phytohormones participate in various processes of plant development (Zhao et al., 2021) and also play important roles in leaf heading formation. Based on RNA-Seq analysis and the crosstalk ability of various phytohormones, five phytohormones (auxin, ethylene, ABA, JA, and GA) were candidates for leafy head formation in Chinese cabbage (Li et al., 2019b). DEGs involved in auxin, ethylene, GA, JA, ABA, BR, CK, and SA signaling pathways play either positive or negative roles in leafy head formation in Chinese cabbage (Yu et al., 2019). The transition leaves of leafy head showed the enrichment of transcripts associated with protein kinases, auxin, and BR pathways and the light-responsive pathway (Wang et al., 2012). Because phytohormones affected leafy head formation, we analyzed the genes with common changes in proteomics and transcriptome of *fg-1* compared with the wild type. We found, in section a, that there are five genes in auxin and ethylene biosynthesis pathway and the JA and ABA signal transduction pathway showed a common change, and in section d, there are eight genes in the JA and ABA biosynthesis pathway, auxin, BA, and SA signal transduction pathway. ABA biosynthesis and signal transduction pathway showed a common change. To clarify the specific role of phytohormones in leaf heading development, it is necessary to further analyze the key genes related to phytohormone biosynthesis and signal transduction.

### 4.4 Key gene affected leaf heading formation

TFs and environmental response genes were believed to play an important role in leaf heading formation in Chinese cabbage (Li et al., 2019b; Wang et al., 2012; Tabusam et al., 2022). In lettuce, loss-of-function of *SAWTOOTH 1* (a kind of TFs) downregulates many adaxial development-related genes but upregulates abaxial development-related genes to control leaf heading formation (An et al., 2022). In Chinese cabbage, *PCF transcription factor4* (*BrpTCP4*) genes modulate the head shape. *CIN-Like TCPs* regulated by temperature dynamics controlled the leaves’ morphological changes in *Arabidopsis* (Malinowski et al., 2011). H^+^-ATPase is affected by environmental stress, which leads to the flat-leaf morphology of *B. rapa* (Zhang et al., 2020). Reports showed that light interacts with auxin during leaf elongation (Fellner et al., 2003). In our study, 86 TFs were exhibited to be co-upregulated and co-downregulated in *fg-1* and WT. Among these, *UDPGT* has four TF candidates, which was one of the genes in the zetin biosynthesis pathway. Besides that, we also found that some of the co-regulated genes were related to photosynthesis, such as *LHCB1* (light-harvesting complex II chlorophyll a/b binding protein 6), *LHCB5*, and *LHCB6*. These indicates that these TFs and light-related genes might regulate leaf heading formation in the early stage, and it needs to be further addressed in the future.

The genes of hormone synthesis and transduction pathway play an important role in leaf heading formation in Chinese cabbage (Li et al., 2019b). Many hormone-related *BrARF3.1* have been reported to affect leaf heading formation (Liang et al., 2016). A screening of the EMS-induced mutant library of Chinese cabbage found that the *GA* biosynthesis-related gene *BrKS1* is involved in leafy head formation (Gao et al., 2020). Both *BrKAO2* and *BrCPS1*, which are involved in the *GA* biosynthetic pathway, have been proven to be responsible for leafy head formation in Chinese cabbage (Gao et al., 2022; Huang et al., 2022), whereas, in this study, we did not find the *GA* biosynthesis-related gene. In this study, 16 genes were found to be involved in plant hormone synthesis and signal transduction pathways by integratedly analyzing quantitative proteomics and transcriptome. Among the 16 genes, in *PYR/PYL*s and *GH3*, the expression level was reported to be altered in the heading and non-heading Chinese cabbage (Li et al., 2019b). Increasing *GH3s* expression levels by overexpression of *OsARF19* (*AUXIN RESPENSE FACTOR19*) in rice reduces IAA content and increases the leaf angle (Zhang et al., 2015). Transcripts of *BcpLH* gene were increased when plants were sprayed with IAA (Yu et al., 2000). In the present study, GH3 transcript and protein co-upregulated in *fg-1*, based on our previous research, and IAA content decreased in *fg-1* (Li et al., 2019b)—maybe *GH3* plays an important role to regulate leafy head formation in Chinese cabbage. The remaining genes are rarely reported to be directly related to leaf heading and require further research.

## 5 Conclusion

The aim of our study was to discover the mechanism of leaf heading formation in Chinese cabbage in the early heading stage. We integratedly analyzed quantitative proteomics with transcriptomics to discover key related genes. A total of 207 and 278 genes were shown to be co-upregulated or co-downregulated in section a and section d, respectively. Based on KEGG analyses, we found that the phenylpropanoid biosynthesis pathway was enriched in both sections a and d. In addition, we further confirmed 86 TFs that were exhibited to be co-upregulated or co-downregulated, and seven out of 86 were involved in plant hormone synthesis and signal transduction pathways. In summary, we have discovered several key early heading-formation-related factors via integratedly analyzing quantitative proteomics with transcriptomics data. This provides potential gene resources for further research on the molecular mechanism of leaf heading formation.

## Data Availability

The authors acknowledge that the data presented in this study must be deposited and made publicly available in an acceptable repository, prior to publication. Frontiers cannot accept a article that does not adhere to our open data policies.
