# Supplementary material for: Integratedly analyzed quantitative proteomics with transcriptomics to discover key genes via fg-1 non-heading mutant in the early heading stage of Chinese cabbage
Source: Front Plant Sci. 2024 Oct 17;15:1467006. doi: 10.3389/fpls.2024.1467006 (PMC11524848; doi:10.3389/fpls.2024.1467006)
Supplement: Supplementary file 1 [file DataSheet1.docx]

**Integratedly analyzed quantitative proteomics with transcriptomics to discover key leaf-heading formation genes in Chinese cabbage**

Jingrui Li, Mi Fan, Xiaomeng Zhang, Liling Yang, Guangguang Hou, Lei Yang, Na Li, Shuxin Xuan^*^, Jianjun Zhao^*^

*Collaborative Innovation Center of Vegetable Industry in Hebei, Hebei Key Laboratory of Vegetable Germplasm Innovation and Utilization, College of Horticulture, Hebei Agricultural University, Baoding, Hebei 071000, China*

*** Correspondence:**Corresponding Author

yyxsx@hebau.edu.cn
jjz1971@aliyun.com

Supplementary Material

# Supplementary Figures


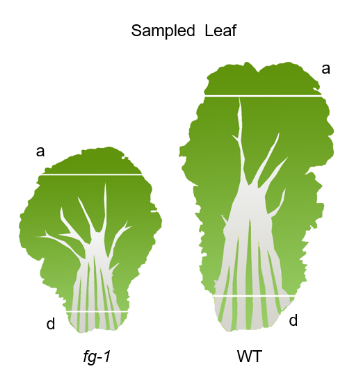


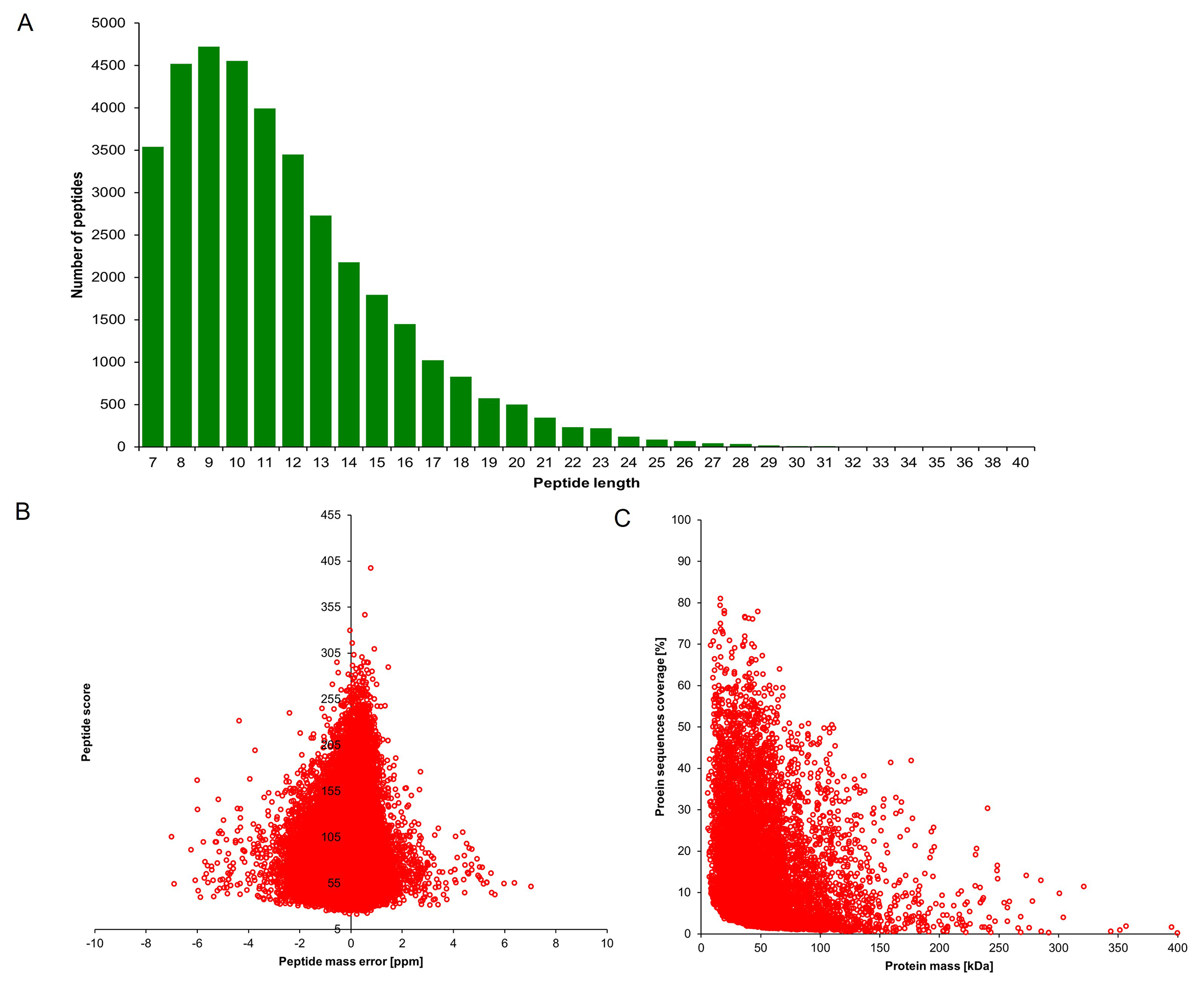
Figure. S1. Schematic diagram of proteome sampling

Figure.S2. Peptide length distribution identified by mass spectrometry (A). Error distribution of spectral quality (B). Relationships between the protein molecular weight and coverage identified by mass spectrometry (C).


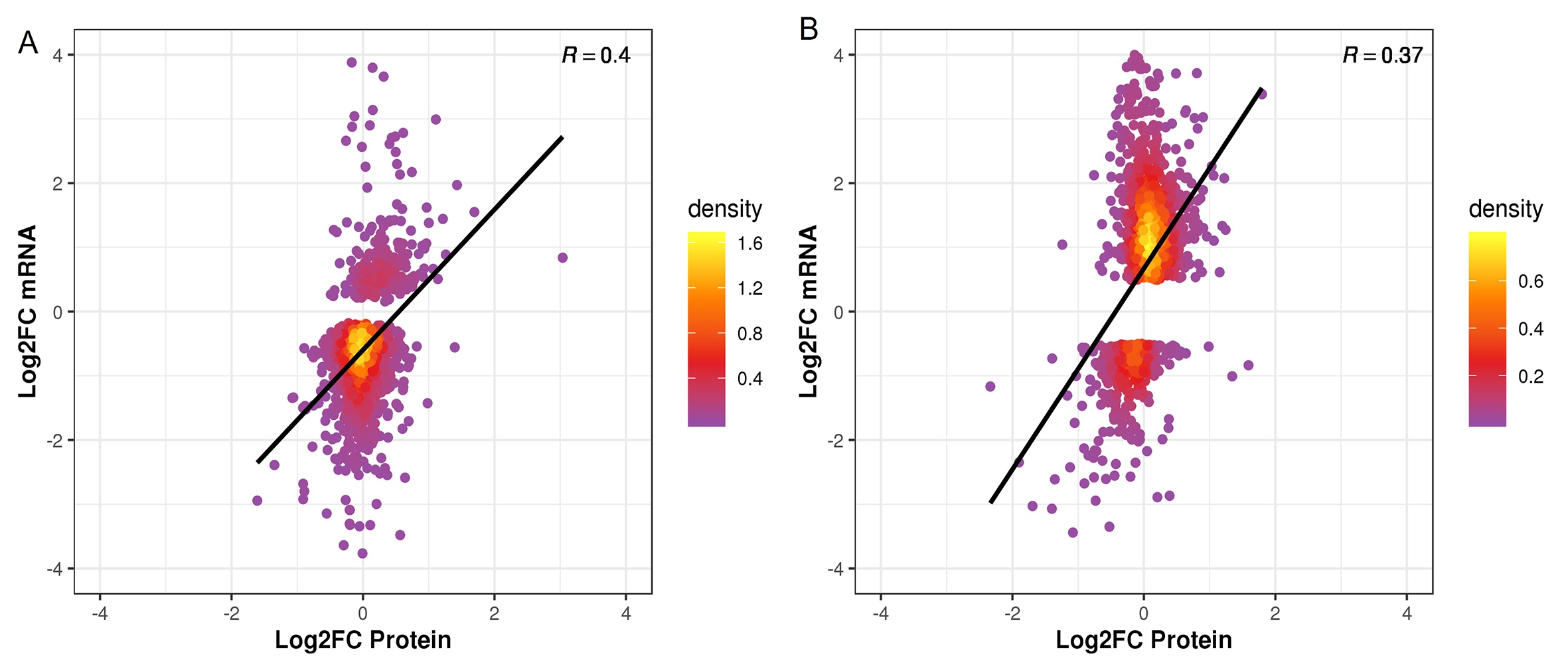


Figure. S3. Scatter plot of the transcripts and their corresponding proteins in the section a (A) and the section d (B) The horizontal axis represents the protein expression levels, and the vertical axis represents the transcript expression levels. The color of the points indicates the density of the points at that location.

Table S1 Basic statistical table of MS results

| Total spectrum | Matched  spectrum | Peptides | Unique  peptides | Identified  proteins | Quantifiable  proteins |
| --- | --- | --- | --- | --- | --- |
| 266694 | 70739 (26.5%) | 37087 | 28081 | 7287 | 5994 |
